# The distribution, diversity, and conservation status of *Cycas* in China

**DOI:** 10.1002/ece3.2910

**Published:** 2017-03-31

**Authors:** Ying Zheng, Jian Liu, Xiuyan Feng, Xun Gong

**Affiliations:** ^1^ Key Laboratory for Plant Diversity and Biogeography of East AsiaKunming Institute of BotanyChinese Academy of SciencesKunmingYunnanChina; ^2^ Key Laboratory of Economic Plants and BiotechnologyKunming Institute of BotanyChinese Academy of SciencesKunmingYunnanChina; ^3^ University of Chinese Academy of SciencesBeijingChina

**Keywords:** conservation biology, *Cycas*, endangered species, fragmentation, genetic variation, population genetics

## Abstract

As ancient gymnosperm and woody plants, cycads have survived through dramatic tectonic activities, climate fluctuation, and environmental variations making them of great significance in studying the origin and evolution of flora biodiversity. However, they are among the most threatened plant groups in the world. The principal aim of this review is to outline the distribution, diversity, and conservation status of *Cycas* in China and provide suggestions for conservation practices. In this review, we describe the taxonomy, distribution, and conservation status of *Cycas* in China. By comparing Chinese *Cycas* species with its relatives worldwide, we then discuss the current genetic diversity, genetic differentiation of *Cycas,* and try to disentangle the potential effects of Quaternary climate changes and topographical events on *Cycas*. We review conservation practices from both researchers and practitioners for these rare and endangered species. High genetic diversity at the species level and strong genetic differentiation within *Cycas* have been observed. Most *Cycas* species in southwest China have experienced population retreats in contrast to the coastal *Cycas*'s expansion during the Quaternary glaciation. Additionally, human activities and habitat fragmentation have pushed these endangered taxa to the brink of extinction. Although numerous efforts have been made to mitigate threats to *Cycas* survival, implementation and compliance monitoring in protection zones are currently inadequate. We outline six proposals to strengthen conservation measures for *Cycas* in China and anticipate that these measures will provide guidelines for further research on population genetics as well as conservation biology of not only cycads but also other endangered species worldwide.

## 1 Introduction

Cycads are the oldest and most primitive assemblages of living seed plants in the world. They originated before the mid‐Permian and reached greatest diversity during the Jurassic–Cretaceous (Jones, 2002; Mustoe, 2007; Nagalingum et al., 2011). However, the current survivors of cycad species are not much older than 12 million years mainly owing to the flourish of flowering plants (Nagalingum et al., 2011). Cycads are essentially “living fossils,” or evolutionary relicts, and are of great scientific and conservation value because of their long evolutionary history. The genetic information contained in cycads is important for paleontology, paleoclimatology, and paleogeography. In addition, cycads are thought to be the earliest gymnosperm lineage (Chaw, Zharkikh, Sung, Lau, & Li, 1997), retaining features which resemble ferns, such as spermatozoa with flagella, and features which belong to spermatophytes, like naked seeds (Guan, 1996). Also, as a taxon that bridges a major evolutionary transition in plants, cycads are indispensable for understanding the origin and subsequent evolution of seed plants. Morphologically, cycads are characterized by a palm‐like habit with stout trunks and crowns formed by large, evergreen and pinnate leaves (Jones, 2002). They are dioecious, entomophilous (e.g., weevils) with their heavy seeds dispersed largely by rodents and small fruit‐eating bats which constrain the level of gene flows from 2 to 7 km (Yang & Meerow, 1996). Fossil records indicate that cycads are once more widely distributed (Wang, Zhang, Zheng, Kc, & Li, 2005); however, the extant taxa are mostly restricted to tropical, subtropical, or warm temperate regions (Jones, 2002). Given their great values in plant evolution and conservation biology, however, no comprehensive literatures about *Cycas* in China have been compiled since 2008 (Hill, 2008).

**Figure 1:**
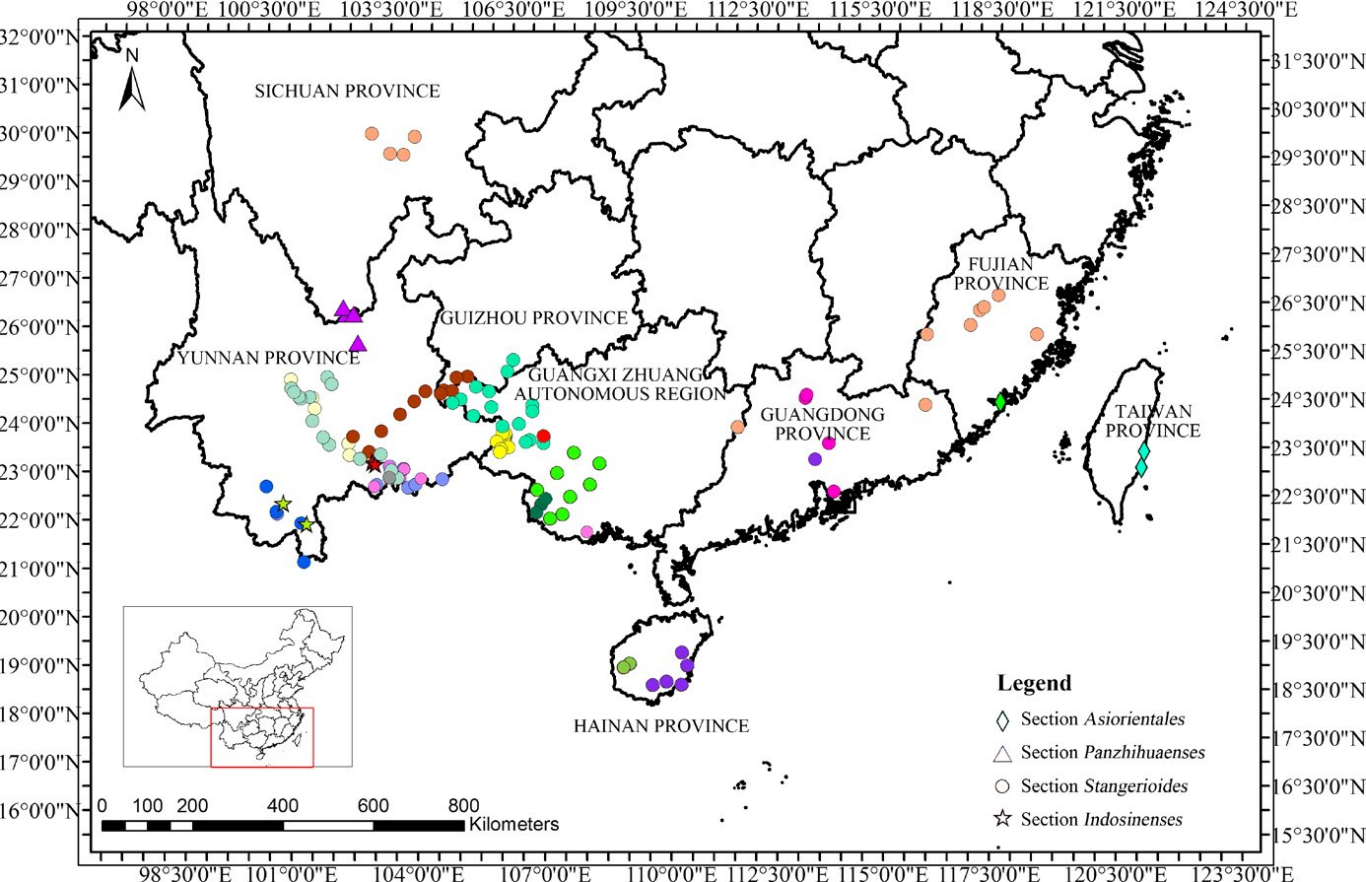
Geographic ranges of 23 *Cycas* species in China. Different shapes represent different sections. The diamonds represent species from section *Asiorientales*. The triangles indicate species from section *Panzhihuaenses*. Species from section *Stangerioides* are marked as circles and species from section *Indosinenses* are signed as starts. Different color represents different species

Apart from conifers, cycads are the most abundant group in gymnosperms including two families (Cycadaceae and Zamiaceae), ten genera, and nearly 348 species (Calonje, Stevenson, & Stanberg, 2017; Christenhusz et al., 2011) scattering in the tropical and subtropical regions with latitude range between 27°S and 18°N (Fragnière, Bétrisey, Cardinaux, Stoffel, & Kozlowski, 2015). The Caribbean and northeast Australia represent the two most diverse floristic regions of cycads with 68 Zamiaceae in Caribbean, 70 *Macrozamia* and *Cycas* species in northeast Australia (Fragnière et al., 2015). As the most widespread genus within extant cycads, *Cycas* occupies several distribution centers with one scattered in China, particularly in the southwestern mountainous regions (Fig. 1). Biogeographic analyses strongly favor a South China origin for the living *Cycas*, with an early dispersal to Indochina (Xiao & Moller, 2015). Because of the habitat heterogeneity and genetic assemblage, there has been a great deal of confusion and debate regarding *Cycas* taxonomy (Hill, 2008; Hill & Stevenson, 1998; Hill, Walters, & Osborne, 2004; Wang, Liang, & Chen, 1996; Wu & Raven, 1999; Zheng & Fu, 1978). The inconsistency derived from morphological and molecular data add extra difficulties in species recognition and delimitation. Until now, 219 names have been proposed for *Cycas*, with 114 accepted, nine regarded as subspecies, 95 synonyms, and one illegitimate name (Calonje et al., 2017).

As a recently radiated lineage, some *Cycas* species in southwest China exhibit high genetic diversity at the species level, low genetic diversity within populations, and significant genetic differentiation among populations (Feng, Wang, & Gong, 2014; Gong et al., 2015; Liu, Zhou, & Gong, 2015; Zheng, Liu, & Gong, 2016). However, specific comparison concerning genetic variations of all the *Cycas* species in China has rarely been performed. In addition, heterogeneous landscapes in southwest China coupled with Quaternary climate fluctuation has left dramatic impacts on the historical dynamic of this lineage. Currently, however, it is the conflict between suitable habitats and human activities that imperil these taxa: Nearly 63% of extant cycads worldwide are on the International Union for Conservation of Nature (IUCN) Red List of Threatened Plants. Most *Cycas* species in Yunnan, China, have been listed as Plant Species with Extremely Small Populations (PSESP) (Ma et al., 2013), displaying the highest proportion of the genus than any other plant groups (15/23, 65%) (Sun, 2013; Yang & Yang, 2014; Zheng et al., 2013). As human disturbance has severely disturbed *Cycas* populations and its habitats, multiple protective efforts have been made over the last decade (Luo, Tang, Huang, Liang, & Zhao, 2014; Zhan, Wang, Gong, & Peng, 2011). However, there are “research‐implementation gaps” between scientific researchers and conservation practitioners hindering the conservation efforts for this endangered taxa (Ottewell, Bickerton, Byrne, & Lowe, 2016).

Considering the great value of cycads in conservation biology and plant evolution comparing with the increasingly endangered status, the inadequate and somewhat out‐of‐date information resources, as well as the inefficient conservation measures, a comprehensive and species‐specific review of *Cycas* in China is in urgent needs. For the foregoing reasons, we retrieve as much literatures, reports, and newspapers as possible to make a systematic description of the taxonomy, distribution, and conservation status of *Cycas* in China. Genetic diversity and genetic differentiation of *Cycas* are analyzed to find out the potential influence of Quaternary climate changes and topographical events on this lineage. Finally, we put forward six suggestions to strengthen *Cycas* conservation in China.

## 2 Current Status of *CYCAS*

### 2.1 The taxonomy of *Cycas*

As the genus *Cycas* was recorded by Linnaeus in 1753, there has been a great deal of confusion and debate regarding *Cycas* taxonomy (Hill, 2008; Hill & Stevenson, 1998; Hill et al., 2004; Wang et al., 1996; Wu & Raven, 1999; Zheng & Fu, 1978). Several factors make *Cycas* taxonomy challenging to resolve: Cycads are dioecious plants with a relatively long period to first coning; strobilus collection is difficult in the wild. Subtle morphological variations between species, caused either by environmental (heterogeneous landscapes or regional microclimate) or genetic factors, reduce the power of traditional taxonomy to delineate species boundaries, such as the *C. segmentifida* D.Y. Wang & C.Y. Deng complex (Chen, Wu, & Raven, 1999; Feng, Liu, & Gong, 2016a; Huang, 2001; Ma, 2005; Wang, 2000; Whitelock, 2002) and the *C. diannanensis* Z.T. Guan & G.D. Tao complex (Liu et al., 2015). Adding to the challenge of species delimitation, incomplete reproductive isolation between *Cycas* and the sympatric distribution of several populations increase the frequency of hybridization in these taxa. Furthermore, new species or subspecies continue to be proposed, while synonyms, illegitimate names, nomina dubia, and invalidly published names are frequently discarded.

Six classification sections are now recognized in the genus *Cycas* (Table 1): four in Hill (1995), an additional one in Lindstrom, Hill, and Stanberg (2008) and another one in Lindstrom, Hill, and Stanberg (2009). According to Hill (2008), 17 species from section *Stangerioides* distributed in China and *C. multifrondis* D. Yue Wang was treated as hybrid swarm of *C. dolichophylla* K.D. Hill, H.T. Nguyen & L.K. Phan, and *C. bifida* (Dyer) K.D. Hill. In Chen, Yang, and Li,'s opinion (2014), however, *C. multifrondis* is treated as an independent species. Results of molecular phylogeography of *C. taiwaniana* Carruth. complex reveal that *C. hainanensis* C.J. Chen is a subspecies of *C. taiwaniana* and *C. shanyaensis* G.A. Fu is not an independent species (Jian & Zhang, 2013). We also suggest that the *C. collina* K.D. Hill, H.T. Nguyen, & L.K. Phan from Xishuangbanna of China is the synonym of *C. simplicipinna* (Smitinand) K.D. Hill based on field surveys and data reference (Hill, 2008). In addition, the newly published species, *C. chenii* X. Gong & Wei Zhou, is affiliated with section *Stangerioides* (Zhou, Guan, & Gong, 2015). Consequently, we propose that there are 18 species occurring in China within section *Stangerioides*. For section *Asiorientales*, two coastal species, namely *C. taitungensis* C.F. Shen, K.D. Hill, C.H. Tsou & C.J. Chen, and *C. revoluta* Thunb., are recognized in China. Section *Panzhihuaenses* contains the sole species from southwest China, that is, *C. panzhihuaensis* L. Zhou & S.Y. Yang*. Cycas pectinata* Buch.‐Ham. and *C. hongheensis* S.Y. Yang & S.L. Yang ex D. Yue Wang are the two Chinese species from section *Indosinenses* (Table 1). In total, 23 species from four sections present in China.

**Table 1:** Key characteristics of six *Cycas* sections worldwide and species list of *Cycas* in China

Sections	Microsporangiate cones and microsporophylls	Megasporophyll apices	Ovules	Seeds	*Cycas* species in China
*Asiorientales*	Firm, waxy	Pectinate	Tomentose	Red seeds with a nonfibrous sarcotesta and a smooth, longitudinally grooved sclerotesta	*C. revoluta*,* C. taitungensis*
*Panzhihuaenses*	Firm, waxy	Pectinate	Glabrous	Red to orange with a nonfibrous sarcotesta and a smooth, unornamented sclerotesta	*C. panzhihuaensis*
*Stangerioides*	Soft	Pectinate	Glabrous	Yellow seeds with a nonfibrous, loose, freely peeling sarcotesta, and a verrucose sclerotesta	*C. balansae*,* C. bifida*,* C. changjiangensis* N. Liu, *C. chenii*,* C. debaoensis*,* C. diannanensis*,* C. dolichophylla*,* C. fairylakea*,* C. ferruginea*,* C. guizhouensis*,* C. multifrondis*,* C. multipinnata*,* C. segmentifida*,* C. sexseminifera*,* C. simplicipinna*,* C. szechuanensis*,* C. taiwaniana*,* C. tanqingii*
*Indosinenses*	Stiff or woody	Deeply pectinate	Glabrous	Orange‐yellow to orange‐red seeds with a layer of fibrous tissue within the sarcotesta, and a smooth sclerotesta	*C. hongheensis*,* C. pectinata*
*Wadeae*	Soft, waxy	Pectinate	Glabrous	Yellow seeds with a nonfibrous sarcotesta and a strongly longitudinally ribbed sclerotesta	
*Cycas*	Woody mature	Entire or dentate (nonpectinate)	Glabrous	Seeds with a spongy layer inside the sclerotesta for subsection *Rumphiae*; Sarcotesta of seed with fibrous layer present for subsection *Cycas*; Sarcotesta lacking both fibrous layer and spongy layer for subsection *Endemicae*	

### 2.2 The distribution of *Cycas*

For the single genus in Cycadaceae, *Cycas* is the most widely spreading group, with representatives reaching as far to Japan and others ranging from Pacific islands, Indochina, northeast Australia to Madagascar, and the east coast of Africa (Jones, 2002). Particularly, section *Asiorientales* is relict and only two closely related species occur in eastern China and southern Japan (Hill, 2008). In China, wild populations of *C. revoluta* were reported to be found in coastal Fujian Province; however, their current distributions need further verification. *Cycas taitungensis* is the only extant species with two endangered populations along the eastern coast of Taiwan (Chiang et al., 2009). There is only a single species included in section *Panzhihuaenses* settling in Sichuan and Yunnan Province of China, namely *C. panzhihuaensis*.

For the section *Stangerioides*, distributions vary from northern Thailand and northeastern Myanmar east to Laos, Vietnam, and southern China. Most Chinese *Cycas* species from section *Stangerioides* scatter in southwest China. Species in section *Indosinenses* ranges from Himalayan India and Nepal east to Vietnam and southern China and south to northern peninsular Malaysia, with a radiation of species in Thailand (Hill, 2008; Hill & Yang, 1999). *Cycas pectinata* and *C. hongheensis* are the two *Cycas* occurring in Yunnan Province of China.

For the remaining two sections, *Wadeae* is a relictual section endemic to Philippines (Lindstrom et al., 2008). The full range of the section *Cycas* is from India and southern Indochina south to Australia and from East Africa east to Tonga (Lindstrom & Hill, 2007; Lindstrom et al., 2009).

### 2.3 The habitat diversity of *Cycas* in China and Indochina

Species of *Cycas* occupies various habitats, from coastal and near‐coastal lowlands to hills. Many species grow in sparse forests and woodlands, a few in grassland, and a substantial number on rocky slopes and escarpments where the vegetation is sparse (Jones, 2002). *Cycas* species in southwest China and Indochina generally grow on low‐altitude slopes of ridges and cliffs along river valleys, their ranges stretching to a relatively low elevation (100–1500 m), for example, *C. panzhihuaensis* distributes along the Jinsha River, *C. diannanensis* and *C. dolichophylla* occur in the Red River region (Fragnière et al., 2015).

The climate of these *Cycas* distributions varies from tropical to subtropical monsoon dominated primarily by hot but humid conditions. Some species, such as *C. tanqingii* D. Yue Wang, *C. multipinnata* C.J. Chen & S.Y. Yang, and *C. segmentifida,* occur in tropical forest, evergreen broadleaf forests as well as secondary forests or bamboo forests with canopy cover. Other species are exposed to open landscapes and limestone habitat characterized by infertile soil condition. *Cycas sexseminifera* F.N. Wei and *C. ferruginea* F.N. Wei grow in steep cliffs or open stone crevices. *Cycas debaoensis* Y.C. Zhong & C.J. Chen grows on a variety of soil types, with the Gula River population occupying sandy areas and other populations scattered to karst type on isolated limestone hills (Zhan et al., 2011).

## 3 Genetics Diversity and Differentiation

### 3.1 Genetic diversity of *Cycas* in China

Genetic variation plays a vital role for endangered species in maintaining their adaptation and viability (Frankham, Briscoe, & Ballou, 2002). A better understanding of genetic diversity and genetic differentiation of *Cycas* in China improves the efficiency of conservation practice. Therefore, to get a precise insight of genetic variations within these lineages, we list genetic diversity and genetic differentiation values of Chinese *Cycas* species with microsatellite (SSR) and chloroplast DNA (cpDNA) data available, and here supplement them with our own unpublished molecular data in Table 2.

**Table 2:** Genetic diversity and population differentiation of *Cycas* in China

Data source	Taxon	*PPL* (%)	*H*e	*G*st/*F*st	Reference(s)
SSR	*Cycas bifida*	89.06	0.5430	0.1156	Gong (2015)
	*Cycas debaoensis*	93.58	0.4840	0.1144	Gong (2015)
	*Cycas dolichophylla*	87.98	0.4660	0.2600^a^	Zheng et al. (2016)
	*Cycas guizhouensis*	88.11	0.4190	0.1380^a^	Feng et al. (2016bb)
	*Cycas hongheensis*	90.00	0.4350	–	Unpublished
	*Cycas multipinnata*	94.12	0.4970	0.2957^a^	Gong et al. (2015)
	*Cycas segmentifida*	84.52	0.4360	0.2290^a^	Feng et al. (2016aa)
	*Cycas simplicipinna*	90.63	0.4470	0.2610	Feng et al. (2014)
	*Cycas szechuanensis*	66.67	0.2470	–	Gong (2012)
	Average	87.19	0.4416	0.2020	
		***H*** **d**	***π***	***G*** **st/** ***F*** **st**	
cpDNA	*Cycas bifida*	0.718	0.0019	0.8328	Gong (2015)
	*Cycas chenii*	0.621	0.0014	0.9540	Yang et al. (2016)
	*Cycas debaoensis*	0.492	0.0013	0.8010	Zhan et al. (2011)
	*Cycas diannanensis*	0.564	0.0009	0.8185	Liu et al. (2015)
	*Cycas dolichophylla*	0.940	0.0025	0.8400^a^	Zheng et al. (2016)
	*Cycas guizhouensis*	0.794	0.0009	0.6982^a^	Feng et al. (2016b)
	*Cycas multipinnata*	0.772	0.0015	0.9230	Gong et al. (2015)
	*Cycas panzhihuaensis*	0.571	0.0038	0.7903	Zhang (2012)
	*Cycas segmentifida*	0.602	0.0023	0.9980^a^	Feng et al. (2016a)
	*Cycas simplicipinna*	0.864	0.0026	0.9867	Feng et al. (2014)
	*Cycas taiwaniana*	0.663	0.0013	0.9160^a^	Jian & Zhang (2013)
	Average	0.691	0.0019	0.8690	
	*Cycas revoluta*	0.959	0.0581	0.0864	Chiang et al. (2009)
	*Cycas taitungensis*	0.998	0.0127	0.0056	Huang et al. (2001)
	Average	0.9785	0.0354	0.0460	

–, Data not available; *PPL*, percentage of polymorphic loci; *H*e, expected heterozygosity; *H*d, haplotype diversity; *π*, nucleotide diversity; *G*st/*F*st, genetic differentiation.

If both *G*st and *F*st were available, chose *F*st in priority.

a *p* < .05, significant value was detected in *F*st.

High level of genetic diversity at the species level was revealed for *Cycas* in China based on SSR data (Table 2). The percentage of polymorphic loci (*PPL*) within the nine reviewed *Cycas* varies from 66.67% to 94.12% with an average value reaching 87.19%. The expected heterozygosity (*H*e) varies from 0.2470 to 0.5430 with an average value about 0.4416. These two parameters reflect dramatically high level of genetic variation of the nine reviewed Chinese *Cycas* when comparing with 655 woody plants (aver. *PPL* = 51.30%, *H*e = 0.150) (Hamrick, Godt, & Sherman‐Broyles, 1992). It is even more remarkable in comparison with six *Cycas* species (aver. *PPL* = 35.6%, *H*e = 0.074) and 20 Zaminaceae species (aver. *PPL* = 62.7%, *H*e = 0.225) that derived from allozymes (González‐Astorga, Vovides, Cabrera‐Toledo, & Nicolalde‐Morejón, 2009). Moreover, this average *PPL* value is higher than a plant species with extremely small populations (*PPL* = 52.80%, *Trigonobalanus doichangensis*) (Sun et al., 2007). *Cycas szechuanensis* W.C. Cheng & L.K. Fu exhibits the lowest values of *PPL* (66.67%) and *H*e (0.2470) which mainly resulted from genetic drift and inbreeding effects giving their small and isolated populations.

At the cpDNA level, high level of genetic diversity is also corroborated (Table 2). The cpDNA haplotype diversity (*H*d) varies from 0.492 to 0.998 for the 13 *Cycas* species with an average value of 0.7352, which is higher than the relict taxon *Ginkgo biloba* (*H*d = 0.1910) (Gong, Chen, Dobeš, Fu, & Koch, 2008). Moreover, most *Cycas* species in China also exhibited higher haplotype diversity than the gymnosperm *Taxus wallichiana* (0.626) (Liu et al., 2013). However, the nucleotide diversity (*π*) is somewhat low. For instance, the average nucleotide diversity index of 11 inland species is 0.0019, which is lower than *Cathaya argyrophylla* (*π* *= *0.0021, Wang & Ge, 2006), but a little higher than *G. biloba* (*π* *= *0.0017, Gong et al., 2008). Noticeably, nucleotide diversity of two coastal or island species, *C. taitungensis* and *C. revoluta,* is an order of magnitude larger than the other 11 inland species (Chiang et al., 2009; Huang, Chiang, Schaal, Chou, & Chiang, 2001).

### 3.2 Level of genetic differentiation for *Cycas* in China

Another general conclusion we found is that significant genetic differentiation among populations exists in *Cycas* from China, especially in southwest China, which is in accordance with results derived from a previous summary of six Cycadaceae species (*F*st = 0.254) (González‐Astorga et al., 2009). High level of genetic differentiation is detected by SSR data (aver. *F*st = 0.2020) and most of cycads analyzed in this study exhibit clear population structure (Table 2). For cpDNA sequences, a significant level of genetic differentiation among populations has been mostly uncovered in inland lineages, such as the cases in *C. segmentifida* (*F*st = 0.9980) and *C. dolichophylla* (*F*st = 0.8400) (Table 2).

In contrast to inland *Cycas* of China, low genetic differentiation among populations is detected in two coastal or island species by cpDNA markers (Table 2). Huang et al. (2001) ascribed this phenomenon to the shared dominant alleles and heterogeneous composition of organelle DNAs within each population. Moreover, the deduced gene flows between populations were extremely high, namely 90.41 in *C. taitungensis* and 10.95 in *C. revoluta*, which might contribute little to estimating current population structure and ongoing gene flow, but likely to represent historical migration events (Chiang et al., 2009).

Based on Bayesian Skyline Plot, a scenario of population expansion was suggested for both *C. revoluta* and *C. taitungensis* (Chiang et al., 2009), which contrary to the contraction experience occurred on southeast Asia *Cycas* species (Feng, Zheng, & Gong, 2016b; Feng, Liu, et al., 2016a; Gong et al., 2015; Zheng et al., 2016). One possible reason for this contrast is the different evolutionary processes of both topography and species in history (Gong et al., 2015; Huang et al., 2001; Kizaki & Oshiro, 1977; Shaw & Huang, 1995).

## 4 Threats to *Cycas*

### 4.1 Human activity

Human activity has long been and continues to be the major threat to species diversity and long‐term survival (Volis, 2016). During the last few decades, the over‐plundering of wild cycad resources for timber, food, medicine, landscaping, and other commercial purposes has resulted in a dramatic decline in *Cycas* species and quantity (Mustoe, 2007; Wang et al., 1996).

Among the 23 species in China, sixteen (69.57%) are listed as threatened by IUCN, six (26.09%) are near threatened, and most are preserved with poor ecological conditions. *Cycas revoluta* and *C. szechuanensis* have nearly gone extinct in the wild due to commercially excessive harvesting. Except for *C. pectinata*, most *Cycas* species occupy quite narrow ranges. For example, four populations of *C. multipinnata* have been discovered in the field with less than 15 individuals per population, and no population of *C. multifrondis* has been recorded in the wild (Personal field investigation).

Additionally, habitat loss or fragmentation caused by human activities has increased *Cycas* survival stress (Terry, Forster, Moore, Roemer, & Machin, 2008). Structural changes to landscapes have been shown to affect plant dispersal patterns, and this in turn ultimately affects community assembly and interactions. Fragmentation can thus lead to the subdivision of a single population into multiple disjunctive subpopulations, finally form small population sizes. However, small populations are susceptible to the loss of genetic diversity, and more easily experience higher extinction rates and changes in population genetics (Kupfer, Malanson, & Franklin, 2006; Ottewell et al., 2016). The conflict between human development and wildlife is ongoing (Santini, Saura, & Rondinini, 2016). Deforestation, pasture, farming, urban expansion, etc. have gradually devoured *Cycas* territory, leaving only isolated pockets, or pushing them out of suitable habitats. Deforestation caused by farming practices represents the biggest threat to *C. hongheensis*. Nowadays, only two *C. hongheensis* populations are found in the wild, with habitats restricted to a dry and hot limestone hill side along the Red River in Yunnan, China.

### 4.2 Climate change

Climate change has been proposed to be particularly threatening to rare plants with narrow distributions, small population sizes, and specific habitat requirements (Ulrey, Quintana‐Ascencio, Kauffman, Smith, & Menges, 2016). During the Quaternary, climatic oscillations have exerted significant influences on genetic diversity and historical dynamics of animals and plants in northern hemisphere by altering species distributions (Qiu, Fu, & Comes, 2011). *Cycas* is no exception to these trends. Most cycads live in valleys or monsoon forests on low‐altitude slopes of ridges and cliffs (Fragnière et al., 2015), where is characterized by warm and moist conditions. However, the glacial–interglacial cycles, accompanied by a strengthened Asian winter monsoon dominating over continental southeast Asia (Cook & Jones, 2012), restricted *Cycas* into scattered habitats and hindered gene flow between populations (Feng et al., 2014; Liu et al., 2015; Xie, Jian, & Liu, 2005; Zhan et al., 2011; Zheng et al., 2016). Consequently, small populations were gradually isolated, which profoundly influenced the current demography, genetic structure, and population size.

More recently, since 1961, southwest China has experienced a 0.7°C rise in temperatures and rainfall has increased by 22–33% (Turkington & Harrower, 2016). Frequent mud‐rock flows and landslides triggered by increased rainfall have ruined habitats. Several field surveys have revealed that only six seedlings of *C. longiconifera* Hung T. Chang, Y.C. Zhong & Y.Y. Huang (now has been recognized as *C. segmentifida*) are found in Guangxi, China (Personal field investigation), where previous records indicate the presence of more populations. Floods have destroyed habitat, drowned individuals, and eroded seeds. Only a small proportion of seeds succeed to germinate. However, their future is worrisome.

## 5 Conservation Efforts

The principal object of conservation biology is to maintain the evolutionary potential of species for their adaptation to the changing environment and prevent them from going extinct (Frankham et al., 2002). As discussed above, it is clear that *Cycas* in China demonstrate a high level of genetic diversity at the species level and strong genetic differentiation among populations. However, human activities, habitat fragmentation, and climate change have largely shrunk the number of populations and their sizes. Additionally, inbreeding depression and genetic drift detected in these taxa call for immediate conservation action.

### 5.1 Conservation practice suggested by scientific researchers

Currently, the conservation plans proposed for cycads mainly focus on *in situ* and *ex situ* conservation. For operational guidelines, Liu et al. (2015) have specified the downstream populations of *C. diannanensis* which occupy high and peculiar haplotypes for prior *in situ* conservation. In addition, *ex situ* conservation and reintroduction measures for many generations have been supplemented for improving the population size and genetic diversity of this endemic and endangered species. For *C. debaoensis*, several measures have been recommended, including setting up nature reserves and protection stations, improving publicity and education, conducting research on fast reproduction, and strengthening government management (Xie et al., 2005). Gong et al. (2015) have also laid out *in situ* and *ex situ* projects for *C. multipinnata*.

### 5.2 Conservation efforts conducted by conservation practitioners

Apart from the instructive recommendations given by researchers, several practical efforts have been carried out by governments (Table 3). All *Cycas* species in China were given first‐grade state protection when *The National Key Protected Wild Plants* was authorized in 1999 (Yu, 1999; Ren et al., 2012). Eleven species of *Cycas* are listed as PSESPs—there are only 120 species listed as PSESP in China (Sun, 2013). While many Chinese *Cycas* species are conserved in nature reserves, others are situated in unprotected environments. Some specific conservation areas have been founded for protecting *Cycas*, such as the National Nature Reserve for *C. panzhihuaensis* in Panzhihua, Sichuan; the National Nature Reserve of Dawei Mountain for protecting *C. hongheensis*,* C. multipinnata,* and *C. diannanensis* in Honghe, Yunnan; and the Nature Reserve for *C. taitungensis* in Taitung, Taiwan (Ma, Li, Su, & Lin, 2005). At the same time, cycads are also protected in other nature reserves. *Cycas guizhouensis* K.M. Lan & R.F. Zou is preserved in Jinzhong Mountain Nature Reserve in Longlin, Guangxi; *C. dolichophylla* is scattered in Gulinqing Nature Reserve and Dawei Mountain Nature Reserve in Yunnan.

**Table 3:** Nature reserves and current situations of *Cycas* in China

Taxon	Nature reserve or protective areas	Current situations observed in the field^b^
*Cycas balansae* complex	Shiwan Mountain National Nature Reserve in Guangxi, China (1^a^)	–
*Cycas bifida*	Chongzuo white‐headed langur National Nature Reserve, Nonggang National Nature Reserve; Provincial Nature Reserve of West Daming Mountain and En City; County Reserve of Chunxiu, Qinglong Mountain, Guangxi, China; Dawei Mountain National Nature Reserve, Yunnan, China (7)	–
*Cycas chenii*	Newly published without any nature reserve	Six natural populations were discovered in Shuangbai and Honghe county, Yunnan, China
*Cycas debaoensis*	Provincial Nature Reserve of Huanglian Mountain and Laohutiao, County Reserve of Gulong Mountain and Defu, Guangxi, China (4)	Three new range records were discovered in Funing county, Yunnan, China. Over‐exploitation with uneven age structure
*Cycas diannanensis*	Dawei Mountain Nature Reserve, Yunnan, China; Nature Reserve in Yuanjiang, State Nature Reserve in dinosaur river, Provincial Reserve of A'mu Mountain in Honghe for *Cycas parvula;* (4) (maybe) Nature Reserve in Dawei Mountain, Yunnan Province for *Cycas multiovula*	This specie has almost extinct in wild with only one population discovered. Most of the remainders are transplanted from adjacent regions; Small population size in Ma street and Huashiban power station resulted from inefficiency management and commercial activity; No wild population was observed
*Cycas dolichophylla*	Provincial Nature Reserve of Gulinqing and Malipo, Yunnan, China (2)	Apart from population in Malipo, other populations are decreasing with population size
*Cycas ferruginea*	City Reserve of Baidong River, Guangxi, China (1)	
*Cycas guizhouensis*	City Reserve of Junzi Mountain in Shizong, Wanfeng Mountain in Luoping; County Reserve of Cuiyun Mountain, Leidaqing in Shizong and Lubuge in Luoping, Yunnan, China. Jinzhou Mountain Nature Reserve in Longlin, Guangxi, China; County Reserve of Qingshui River, Xianheping and Karst landscape in Pogang, Sichuan, China (9)	Severe dwindling in population size with individuals in field less than 50 per population
*Cycas hainanensis*	Wuzhi Mountain National Nature Reserve, Provincial Reserve of Yinggeling, Ganshiling; City Reserve of Sanya mangrove, Hainan, China (4)	–
*Cycas hongheensis*	Dawei Mountain Nature Reserve, Gejiu County Reserve, Yunnan, China (2)	Only two populations remained with individuals <1,000. Cones were first observed in 2015
*Cycas longipetiolula*	Nature Reserve in Dawei Mountain, Yunnan, China (1)	Only cultivated plants were discovered in Jinping, Yunnan, China
*Cycas multifrondis*	Nature Reserve in Dawei Mountain, Yunnan, China (maybe)	No wild population was recorded.
*Cycas multipinnata*	Natural Reserve in Dawei Mountain, Yunnan, China (1)	Four populations were discovered in field with less than 15 individuals per population
*Cycas panzhihuaensis*	National Nature Reserve for *Cycas panzhihuaensis* in Panzhihua, Provincial Reserve of Liangshan, Sichuan, China; Jiaozi Snow Mountain National Nature Reserve, Provincial Reserve in Luquan, City Reserve in Yuanmo, Yunnan, China (5)	Species in nature reserve keep in healthy growth, while populations in Luquan, Yunnan, China, is shrinking
*Cycas pectinata*	Naban River National Nature Reserve, Xishuangbanna Nature Reserve, Nuozhadu Provincial Nature Reserve in Yunnan, China (3)	–
*Cycas segmentifida*	County Reserve for cycads in Wangmo, Guizhou, China; Cenwanglaoshan Nature Reserve, Yachang Orchids Nature Reserve; Provincial Reserve of Dahongbao, Dawangling and Wangzi Mountain, Guangxi, China (7)	Most of these taxa kept in ideal status, however, no more than 30 plants were observed in BanBang village
*Cycas sexseminifera*	Daming Mountain National Nature Reserve, Chongzuo white‐headed langur National Nature Reserve, Nonggang National Nature Reserve; Provincial Reserve of thirty‐six lane‐Longjun, West Daming Mountain, Xialei, En City; County Reserve of Chunxiu, Qinglong Mountain, Guangxi, China (9)	–
*Cycas simplicipinna*	Naban River National Nature Reserve, Xishuangbanna Nature Reserve, Nuozhadu Provincial Nature Reserve in Yunnan, China (3)	Habitat fragmentation
*Cycas szechuanensis*	Cultivated in Fuhu tempale in E'mei, Sichuan, China	No wild population was recorded. Only cultivated female plants in Sichuan, Guizhou, Fujian, and Yunnan Province.
*Cycas taitungensis*	Nature Reserve for *Cycas taitungensis* in Taiwan (1)	–
*Cycas tanqingii*	Huanglian Mountain National Nature Reserve in Lvchun, Yunan, China (1)	A relative healthy population benefit from early conservation and long‐term monitoring

–, Data not available.

a Number of nature reserves or protective areas for *Cycas* in China.

b Current situations of *Cycas* in the field were observed by our research group during samplings for molecular research.

Apart from these nature reserves, botanical gardens have also contributed to the endeavor. South China Botanical Garden was the first organization to conduct *ex situ* conservation for cycads, with 43 species belonging to nine genera currently planted (Huang, 2014). A nature conservation center for cycad germplasm resources was established by Shenzhen Fair Lake Botanical Garden (SZBG) in 2002. To date, nearly 240 species embracing two families and ten genera have been collected. A reintroduction plan initiated by the State Forestry Administration of China and SZBG for *C. debaoensis* was implemented in 2007 (Yin, 2011). According to a follow‐up study, the reintroduced seedlings in Huanglian Mountain Nature Reserve in Debao county, Guangxi, have started flowering and seed setting (Gan et al., 2013). The China Germplasm Bank of Wild Species based at Kunming Institute of Botany houses 8,855 species (Volis, 2016). However, only the seeds of *C. panzhihuaensis* are gathered in this bank.

## 6 Future Challenges and Conservation Suggestions

Although much progress in *Cycas* conservation has been made in recent years, conservation efforts still suffer from an inadequate conceptual foundation and a lack of systematic planning (Hurka, 1994). Moreover, decentralized management and poor coordination, as well as an absence of clear boundaries, specific management teams, and staff (Liu et al., 2003), call for further actions to preserve this endangered lineage from extinction. Apart from these, pests (e.g., cycad *Aulacaspis*) introduced by horticultural transplantation have jeopardized native species (Cibrian‐Jaramillo, Daly, Brenner, Desalle, & Marler, 2010). Based on the status quo and detailed conservation applications proposed by previous cycads researchers, we put forward the following six recommendations.

### 6.1 Habitat protection and restoration

Suitable habitat/niche is the prerequisite for species to survive, especially for PSESPs. As mentioned above, the major factors that imperil *Cycas* populations come from habitat destruction, whether induced by human activities or fragmentation. Thus, habitat protection and restoration should be given priority in *Cycas* conservation. The practice of habitat restoration includes erosion control, reforestation of disturbed areas, reintroduction of native species, removal of invaders, selectively clearing aggressive native plants, as well as soil amendments (Rey Benayas, Newton, Diaz, & Bullock, 2009). Within the nature reserves, any farmland or pasture should be returned to natural conditions. Poaching rare and endangered plants should not be allowed. As debris flow is the major threat for *C. segmentifida* population in Guangxi, erosion control and reforestation of native species should be carried out to recover the habitat. However, translocation can also be considered as an alternative once the habitat is no longer suitable for such a small population.

### 6.2 *In situ* conservation

New reserves should be created for populations with a considerable number of individuals currently located in unprotected areas. Small‐scale reserves or plant micro‐reserves (PMR; Laguna, 2001; Laguna et al., 2004) have long been recognized as an efficient way to protect small populations in a fragmented landscape (Cowling, Pressey, Rouget, & Lombard, 2003; Draper, Rosselló‐Graell, Garcia, Gomes, & Sérgio, 2003; Götmark & Thorell, 2003; Kadis, Thanos, & Lumbreras, 2013). Of course, small reserves are not an alternative to large nature reserves, but a complement to them, being the only option available to protect natural fragments surrounded by land unsuitable for conservation.

Field investigations conducted by our research group in Yunnan, China, revealed a new population of *C. segmentifida*. The population size is large (>2,000 individuals) and the population structure is healthy with a third of plants are juveniles. Therefore, a new reserve plot for this species is needed *in situ*. Appropriate management plans should also be implemented immediately to maintain its population structure, including seed collection, population reinforcement, pest and disease control, scrub clearance, and habitat restoration. Reinforcement, that is, augmentation of existing populations to enhance population viability (IUCN/SSC, 2013), is another option for species whose remaining populations are located in protected and nondegraded areas (see Soorae, 2016 for example). The material for reinforcement must originate either from the same location or from the geographically closest populations within the same habitat, as well as being genetically diverse.

### 6.3 *Ex situ* conservation

Habitat fragmentation and environmental degradation reduce population viability below the threshold. Thus, *ex situ* conservation of threatened species requires identifying the habitats in which viable populations can be maintained and then protecting both the habitats and the species through carefully designed management (Volis, 2016). For populations that are extinct in the wild or located in unprotected and rapidly deteriorating environments, reintroduction, that is, placement of plant material into an area where it occurred in the past, is a highly valued approach. Translocation, that is, movement of plant material to a seemingly suitable area with no documented past history of its existence, is the conservation action used to prevent species extinction when there is no remaining area left within a species’ historic range able to sustain viable populations (Hoegh‐Guldberg et al., 2008). The successful reintroduction of *C. debaoensis* in Huanglian Mountain Nature Reserve has set a good example for *ex situ* conservation practice (Gan et al., 2013). In both reintroduction and translocation, creation of viable new populations requires prior knowledge of the species biology. Specifically, during *ex situ* conservation, genetic variation within the protected species should be considered in order to enhance the survival ability during long‐term protection.

### 6.4 Construct plant resource banks

In order to meet social needs and relieve cycads of the threat of extinction, it is feasible to construct plant resource banks, such as germplasm repositories, seedling nurseries, experiment, and demonstration zones. Moreover, cultivation should also be promoted and encouraged. During these actions, a key problem is derived from the long vegetative period. One solution is to establish seedling nurseries via seeds because seedlings are more effective for introduction compared with seeds (Godefroid et al., 2011; Guerrant & Kaye, 2007). In addition, experimental research that facilitating seed germination, seedling survival and vegetative propagation should also be conducted and applied in practice. For germplasm repositories, resources should be gathered from different lineages to preserve as much genetic diversity as possible.

### 6.5 Dissemination and education

Protecting species *in situ* and *ex situ* are efficient approaches, but conservation efforts should not be limited to these alone. Education and dissemination should also be improved, especially in local communities where the most dominant reason of ongoing wild species loss is the lack of awareness or appreciation of the true value these species possess beyond their practical worth (Volis, 2016). For the indigenous communities, promoting and popularizing science education should be carried out to improve their awareness of the importance of plant conservation.

If possible, efforts should be made to convert local people into allies in the conservation of cycads that would be a powerful strength. For gardeners, breeding technologies should be promoted to improve cycad survival and adaptability. For forest rangers and volunteers, professional training should be facilitated to improve their management skills.

### 6.6 Construction of a global network for international communication

Information globalization and communication is a prerequisite for efficient conservation of threatened species. The World List of Cycads ( http://cycadlist.org) is a worldwide database providing a comprehensive taxonomic reference for cycads. The IUCN Red List of Threatened Species ( http://www.iucnredlist.org) is another database for cycads where information is collected about taxonomy, assessment, geographic range, population, habitat and ecology, as well as threats and conservation actions. Although most *Cycas* (98 species) are listed by IUCN, the information these networks have released is inadequate and some recently named species are not included. For example, *C. chenii* is a newly published species with six natural populations discovered in Yunnan, China (Zhou et al., 2015). However, this species is not listed by the IUCN and no protective action has been implemented.

Additionally, some information is out of date, that is, the current status is far more critical than listed on the IUCN website. Such is the case for *C. diannanensis*, whose population size is thought to be less than 5,000 mature individuals and continues to decline according to the IUCN. Nevertheless, based on our field survey, this species is almost extinct in the wild, where there is only one population remaining, with less than 2,000 individuals discovered. Poor information sharing due to an inadequate system for disseminating/obtaining information significantly hinders conservation of cycads.

One recommendation is to establish a cycad‐specific comprehensive platform for long‐term monitoring, records, and information sharing. The content of wild monitoring includes population structure, habitats, threats, and protective status. Moreover, species management‐related manuscripts, reports, and expert opinions should also be collected and deposited.

## 7 Conclusions

For the second largest assemblage of gymnosperms, cycads have received far less attention than other gymnosperms (Cun & Wang, 2010; Fragnière et al., 2015; Gong et al., 2008; Li et al., 2005; Sun, Li, Li, Zou, & Liu, 2015). Phylogeny, systematics, and ecology are among the disciplines that are most heavily represented in recent cycad literature. However, considering the fact that nearly 70% of Chinese *Cycas* taxa are listed as threatened, most of them lack applied research which hindering the conservation efforts. Therefore, after a comprehensive literature synthesis and analysis, we have proposed six recommendations for *Cycas* conservation in China. We hope that with the joint efforts of researchers, governments, and the public, the future for *Cycas* will be promising. Apart from *Cycas*, we also expect this study will inspire more work on population genetics and conservation applications for other rare and endangered species in the world.

## Conflict of Interest

None declared.
